# TNFα Enhances Tamoxifen Sensitivity through Dissociation of ERα-p53-NCOR1 Complexes in ERα-Positive Breast Cancer

**DOI:** 10.3390/cancers13112601

**Published:** 2021-05-26

**Authors:** Hyunhee Kim, Seung-Ho Park, Jangho Lee, Gi-Jun Sung, Ji-Hye Song, Sungmin Kwak, Ji-Hoon Jeong, Min-Jeong Kong, Jin-Taek Hwang, Hyo-Kyoung Choi, Kyung-Chul Choi

**Affiliations:** 1 Asan Medical Center, Department of Biomedical Sciences, AMIST, University of Ulsan College of Medicine, Seoul 05505, Korea; ttlok1816@mail.ulsan.ac.kr (H.K.); mylove9322@mail.ulsan.ac.kr (S.-H.P.); iamassong@mail.ulsan.ac.kr (J.-H.S.); bigawa88@mail.ulsan.ac.kr (S.K.); max1431@mail.ulsan.ac.kr (J.-H.J.); kmj0921@mail.ulsan.ac.kr (M.-J.K.); 2 Korea Food Research Institute, Wanju-gun 55365, Korea; jhlee@kfri.re.kr (J.L.); jthwang@kfri.re.kr (J.-T.H.); 3 Department of Obstetriccs, Gynecology and Reproductive Biology, Michigan State University, East Lansing, MI 49534, USA; gjsung89@msu.edu; 4 Department of Food Biotechnology, Korea University of Science and Technology, Daejeon 34113, Korea

**Keywords:** ER-positive breast cancer, tamoxifen resistance, TNFα, *NCOR1*

## Abstract

**Simple Summary:**

Tamoxifen has been clinically applied as a central chemotherapeutic agent for treatment of estrogen receptor (ER)-positive breast cancer. However, many ER-positive breast cancer patients with the high ER level demonstrate intrinsic resistance against the tamoxifen therapy. The aim of our study was to find an effective approach to enhance tamoxifen sensitivity. We found that tumor necrosis factor α (TNFα) has a potential to overcome tamoxifen resistance through disruption of nuclear receptor corepressor 1 (NCOR1)-p53-ERα complexes in ER-positive MCF7 xenograft mice. *NCOR1* knock-down with TNFα treatment induced ERα destabilization and increased the occupancy of p53 at the *p21* promoter. Finally, we confirmed the combinational application with tamoxifen, TNFα and short-hairpin *NCOR1* showed the enhanced suppressive effect of tumor growth in MCF xenograft mice compared to single tamoxifen treatment. These results provide a possibility for application of NCOR1 as a putative therapeutic target to overcome tamoxifen resistance in ERα-positive breast cancer.

**Abstract:**

Tamoxifen is widely used as a medication for estrogen receptor α (ERα)-positive breast cancer, despite the ~50% incidence of tamoxifen resistance. To overcome such resistance, combining tamoxifen with other agents is considered an effective approach. Here, through in vitro studies with ER-positive MCF7 cells and ER-negative MDA-MB-231 cells, validated by the use of xenograft mice, we investigated the potential of tumor necrosis factor α (TNFα) to enhance tamoxifen sensitivity and identified NCOR1 as a key downstream regulator. TNFα specifically degraded nuclear receptor corepressor 1 (NCOR1) in MCF7 cells. Moreover, knockdown of *NCOR1*, similar to TNFα treatment, suppressed cancer cell growth and promoted apoptosis only in MCF7 cells and MCF7 xenograft mice through the stabilization of p53, a tumor suppressor protein. Interestingly, *NCOR1* knockdown with TNFα treatment increased the occupancy of p53 at the *p21* promoter, while decreasing that of ERα. Notably, NCOR1 formed a complex with p53 and ERα, which was disrupted by TNFα. Finally, combinatorial treatment with tamoxifen, TNFα and short–hairpin (sh)-NCOR1 resulted in enhanced suppression of tumor growth in MCF7 xenograft mice compared to single tamoxifen treatment. In conclusion, TNFα promoted tamoxifen sensitivity through the dissociation of the ERα-p53-NCOR1 complex, pointing at NCOR1 as a putative therapeutic target for overcoming tamoxifen resistance in ERα-positive breast cancer.

## 1. Introduction

Breast cancer is the most frequently diagnosed cancer and ranks second among cancer-related death causes in women [1]. Breast cancer, a complex and heterogeneous disease, typically shows variable response to therapies and is characterized by various cancer subtypes displaying significantly different outcomes [2,3]. Traditionally, genetic, epigenetic, environmental, and stochastic factors are believed to contribute to the intratumor heterogeneity of breast cancer, which may lead to therapeutic resistance and thus presents a major obstacle to a cure [4]. Therefore, a better understanding of breast cancer pathogenesis can help develop more effective treatments for this disease.

The expression of estrogen receptor (ER) in breast cancer is one of the most representative heterogeneity indexes. Indeed, ER-negative breast tumors exhibit higher genomic instability and more variation in differential regions with respect to ER-positive tumors [5]. Approximately 75% of breast cancers express ERα, which remains the most important prognostic factor for the effectiveness of endocrine therapy in breast cancer [6,7]. Indeed, drugs either inhibiting estrogen production or targeting the estrogen-binding domain of ERα are the mainstay of the therapies against ERα-positive breast cancer [8,9]. In particular, tamoxifen, a selective antagonist of ER, is the most commonly used medication for the treatment of ERα-positive breast cancer [10]. Tamoxifen contributes to the reduction of breast cancer mortality by 30%; however, about half of ERα-positive breast cancer patients display intrinsic resistance to tamoxifen during treatment [11]. Thus, tamoxifen resistance remains a major hurdle for cancer chemotherapy in ERα-positive breast cancer.

To overcome tamoxifen resistance, combinatorial treatment with tamoxifen and other drugs has emerged as a promising approach. Indeed, drugs targeting molecular pathways involved in tamoxifen resistance, such as the receptor tyrosine kinase, PI3K-mTOR-AKT [12], and cell cycle checkpoint pathways [13], are considered valuable agents for combination with tamoxifen based on preclinical evidence. In our recent study, we demonstrated that tumor necrosis factor α (TNFα), a multifunctional inflammatory cytokine, selectively triggers apoptotic cell death in ERα-positive, but not ERα-negative, breast cancer. In fact, TNFα induces ERα degradation, thereby impairing the localization of ERα on the p53-binding site of the *p21* promoter, and resulting in transcriptional activation of *p21* [14]. However, the transcriptional activity of ER is also modulated by interactions with co-regulatory proteins that function either as co-activators or co-repressors [15]. For instance, the nuclear receptor corepressor 1 (NCOR1) is a well-known co-repressor of nuclear receptors and many other transcription factors [16]. Interestingly, previous studies demonstrated that the level of *NCOR1* mRNA is correlated with a significantly shorter relapse-free survival [17] and with the ERα status of breast cancer patients [18]. Thus, we hypothesized that NCOR1 is involved in TNFα-mediated ERα repression and subsequent p53-dependent activation of *p21* expression in ERα-positive breast cancer.

To verify this hypothesis, in the present study we examined the specific effect of TNFα on the promotion of tamoxifen sensitivity in ERα-positive breast cancer and identified NCOR1 as a key regulator acting downstream of TNFα.

## 2. Results

### 2.1. NCOR1 Knockdown Suppresses the Growth of MCF7 Cells but Not of MDA-MB-231 Cells In Vitro and in Xenograft Mouse Models

**Figure 1:**
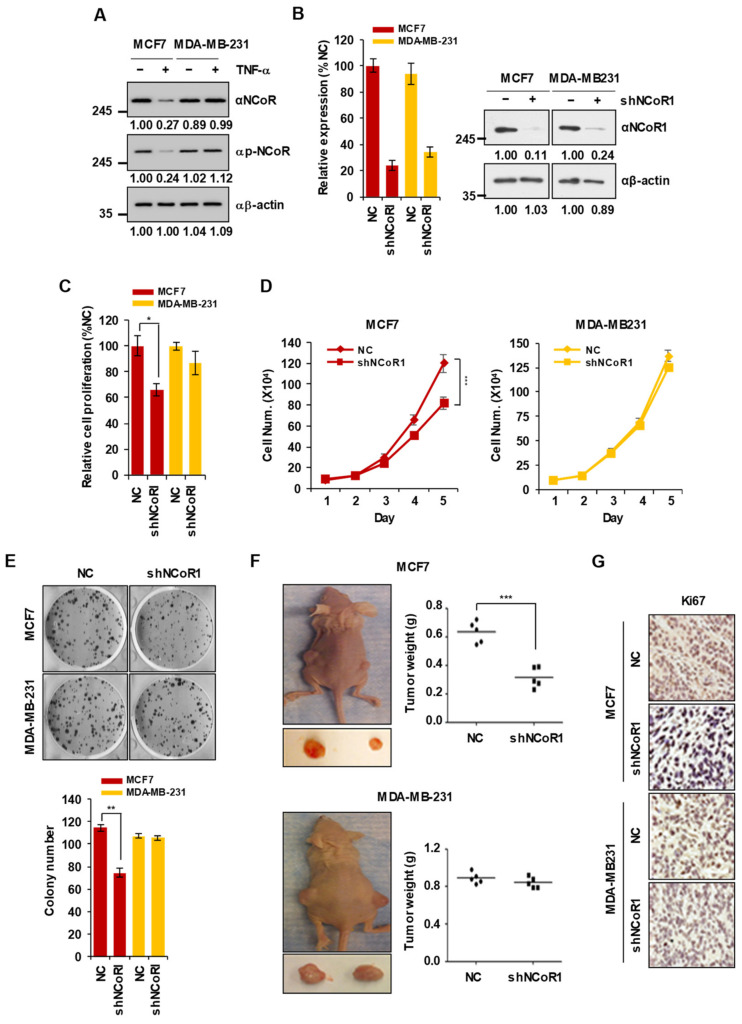
*NCOR1* knockdown selectively suppresses the proliferation of MCF7 cells but not of MDA-MB-231 cells in vitro and xenograft mouse models. (**A**) NCOR1 levels are diminished in presence of TNFα in MCF7 cells but not in MDA-DB-231 cells. The levels of NCOR1 or phospho-NCOR1 (p-NCOR1) were monitored in either MCF7 or MDA-MB-231 cells after TNFα treatment for 24 h. Whole-cell lysates were immunoblotted with the indicated antibodies. Intensities of protein bands obtained from the immunoblotting assay were quantified with ImageJ and normalized with respect to that of β-actin. Relative intensity was calculated by the normalized control intensity of each protein. (**B**) Validation of *NCOR1* knockdown in MCF7 and MDA-MB-231 cells. *** *p* < 0.001; Student’s *t*-test. Relative intensity was calculated as described above (Figure 1A). (**C**,**D**) Silencing of *NCOR1* reduces the proliferation of MCF7 cells. * *p* < 0.05; Student’s *t*-test (**C**). *** *p* < 0.001; Two-way ANOVA (**D**). (**E**) NCOR1 is involved in the malignant transformation of MCF7 cells. 1 × 10^3^ of sh-Control or sh-NCOR1-injected cells were seeded into agarose-coated plates with agarose. Three weeks after incubation, plated cells were stained with crystal violet (upper panel) and the number of colonies was counted (lower panel). Representative images of three independent experiments are shown. ** *p* < 0.01; Student’s *t*-test. (**F**,**G**) In vivo validation of NCOR1-mediated suppression of tumor growth. Stable cell lines transfected with sh-Control or sh-NCOR1 were implanted into the subcutaneous space of the left or right flank of mice, respectively. All tumors were collected 3 weeks after inoculation for determination of tumor weight and volume by necropsy (*n* = 5 for each group). *** *p* < 0.001; Student’s *t*-test (**F**). Representative images of Ki67 immunostaining of tumor tissues (**G**). The values presented are the means ± SD of three independent experiments.

Our recent study reported that TNFα selectively induces apoptotic cell death in ERα-positive but not ERα-negative breast cancer [14]. However, NCOR1 is known as an ERα transcriptional repressor [19]. Thus, we assumed that NCOR1 modulates different responses to TNFα in ERα-positive or -negative breast cancer cells. To verify this hypothesis, we investigated whether the dynamics of NCOR1 protein levels upon TNFα treatment differed between ER-positive MCF7 breast cancer cells and ER-negative MDA-MB-231 cells. Interestingly, the levels of NCOR1 and its phosphorylation, resulting in protein stabilization, were reduced by TNFα treatment in MCF7 cells but not in MDA-MB-231 cells (Figure 1A). Next, to determine whether NCOR1 affects the proliferation of breast cancer cells, we transfected MCF7 and MDA-MB-231 cells with short-hairpin RNAs (shRNAs) targeting *NCOR1* (sh-NCOR1). After demonstrating the successful introduction of sh-NCOR1 into both MCF7 and MDA-MB-231 cells (Figure 1B), cell proliferation and colony formation assays were carried out. Notably, knockdown of *NCOR1* significantly reduced the viability of MCF7 cells (*p* < 0.05) but did not affect that of MDA-MB-231 cells (Figure 1C). Moreover, cell growth was significantly suppressed from 4 days after *NCOR1* knockdown in MCF7 cells (*p* < 0.001) (Figure 1D, left panel) but not MDA-MB-231 cells (Figure 1D, right panel). Finally, *NCOR1* knockdown also selectively inhibited colony formation in MCF7 but not in MDA-MB-231 cells (*p* < 0.01) (Figure 1E). To examine whether NCOR1 affects breast cancer development in vivo, we generated xenograft mouse models by injecting either parental or *NCOR1*-silenced MCF7 or MDA-MB-231 cells to the subcutaneous space of immunocompromised mice, and measured tumor weight 3 weeks after inoculation. Interestingly, tumor weight was significantly reduced in the xenograft mouse model inoculated with *NCOR1*-silenced MCF7 cells (*p* < 0.001) (Figure 1F, upper panel), whereas *NCOR1* knockdown did not significantly reduce tumor weight in MDA-MB-231 xenograft mice (Figure 1F, lower panel). Furthermore, immunostaining for the cell proliferation marker Ki67 also showed that *NCOR1* knockdown selectively inhibited tumor cell proliferation in MCF7 but not MDA-MB-231 xenograft mice (Figure 1G). Collectively, these results suggest that NCOR1 levels are specifically decreased by TNFα and that this co-repressor positively regulates cell growth in ERα-positive but not ERα-negative breast cancer.

### 2.2. NCOR1 Knockdown and TNFα Treatment Exert Similar Apoptosis-Inducing Effects in MCF7 Cells or Tumor-Suppressing Effects in MCF7 Xenograft Model

**Figure 2:**
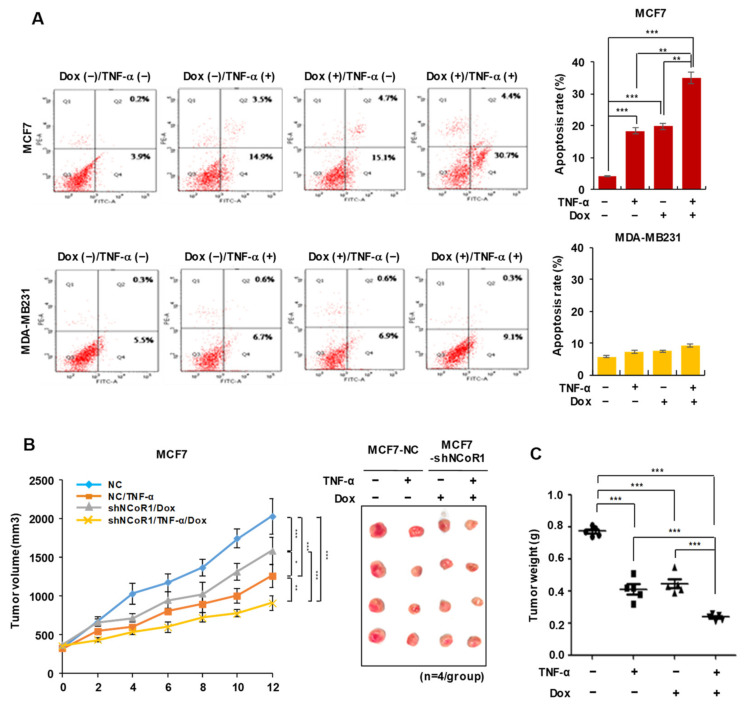
TNFα or *NCOR1* knockdown specifically induces apoptosis in MCF7 cells and inhibit tumor growth in MCF7 xenograft models. (**A**) Apoptotic cell death is increased in response to TNFα in *NCOR1*-silenced MCF7 cells. Doxycycline (Dox)-inducible system for silencing of *NCOR1* was established and applied to MCF7 or MDA-MB-231 cells. After treatment with TNFα for 24 h, the proportion of apoptotic cells was assessed by flow cytometry. Q1; necrotic cell death, Q2; late apoptotic cell death, Q3; control, and Q4; early apoptotic cell death (upper panel). The sum of the ratio of early and late apoptotic cells is shown as the total proportion of apoptotic cells (lower panel). ** *p* < 0.01 and *** *p* < 0.001; Student’s *t*-test. (**B**,**C**) Knockdown of *NCOR1* retards the development of xenograft tumors derived from MCF7 cells. Dox-inducible knockdown of *NCOR1* exerts a synergistic effect on the repression of tumor growth by TNFα. MCF7 cells expressing sh-Control or sh-NCOR1 were implanted into the subcutaneous space of mice, and then Dox or/and TNFα were administered every other day until necropsy. Tumor volumes were calculated every 2 days for 12 days, and representative images were captured. Data are expressed as mean ± SD (*n* = 5 for each group). * *p* < 0.05, ** *p* < 0.01, and *** *p* < 0.001; Two-way ANOVA (**B**). All tumors were removed for necropsy, during which tumor weight was measured. All data are expressed as means ± SD (*n* = 5 for each group). *** *p* < 0.001; Student’s *t*-test (**C**). The values presented are the means ± SD of three independent experiments.

To further examine whether NCOR1 is involved in the specific TNFα-induced apoptosis of ER-positive breast cancer cells, we established doxycycline (Dox)-inducible knockdown of *NCOR1* in either MCF7 or MDA-MB-231 cells. Next, the ratio of apoptotic cells was examined using fluorescence-activated cell sorting (FACS). Notably, the proportion of apoptotic cells in either TNFα-treated or *NCOR1*-silenced MCF7 cells was approximately 20%, higher than that of the control group, but there was no significant difference between these two groups. Interestingly, co-treatment with TNFα and Dox resulted in enhanced apoptotic cell death with respect to TNFα or Dox treatment alone (*p* < 0.01) (Figure 2A, upper panel). On the contrary, *NCOR1* knockdown, TNFα treatment, or their combination did not induce apoptosis in MDA-MB-231 cells (Figure 2A, lower panel). To further evaluate whether NCOR1 depletion, similar to TNFα, induces apoptotic cell death in vivo, we conducted Dox-induced knockdown of *NCOR1* in the MCF7 xenograft mouse model. Tumor volume was monitored every 2 days for 12 days and was found to significantly decrease from 4 days in TNFα-, Dox-, or co-treated groups compared to the control group (*p* < 0.05). Additionally, TNFα- and Dox-treated groups showed no significant difference in tumor volume from 4 days to 12 days except at 10 days (Figure 2B). At 12 days, the mice were sacrificed, and the weight of the tumors isolated from the xenograft was measured. Tumor weight was significantly reduced by TNFα, Dox, or their combination (*p* < 0.001). Notably, no significant difference between TNFα-treated and *NCOR1*-silenced groups was observed (Figure 2C). Therefore, these results suggest that NCOR1 is a key regulator of TNFα-induced apoptosis and tumor suppression in ER-positive breast cancer.

### 2.3. NCOR1 Knockdown Induces p53 Stabilization upon TNFα Treatment in MCF7 Cells and MCF7 Xenograft Mice

**Figure 3:**
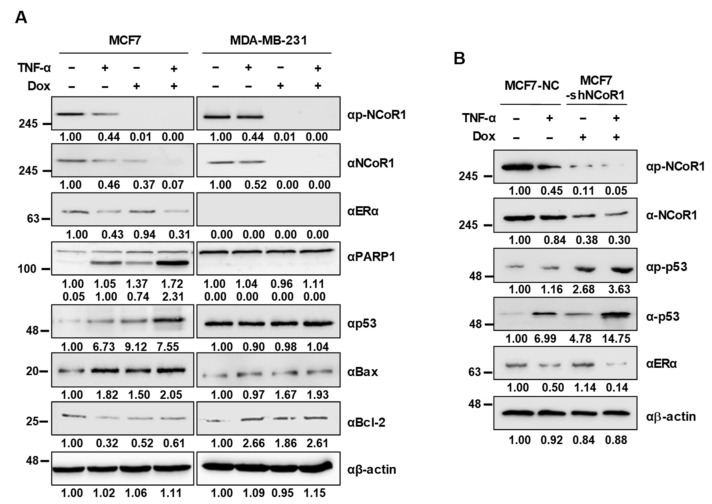
TNFα treatment or *NCOR1* knockdown stabilize p53 in MCF7 cells and tumors from MCF7 xenograft mice. (**A**) p53 stabilization was specifically induced by TNFα or *NCOR1* knockdown in MCF7 but not MDA-MB-231 cells. Doxycycline (Dox) or/and TNFα were administered either to MCF7 or MDA-MB-231 cells, and whole-cell lysates were obtained. Proteins were immunoblotted with the indicated antibodies. Intensities of protein bands obtained from the immunoblotting assay were quantified with ImageJ and normalized with respect to that of β-actin. Relative intensity was calculated by the normalized control intensity of each protein. (**B**) p53 stabilization was induced by TNFα or *NCOR1* knockdown in MCF7 xenograft mice. Tumors were isolated from MCF7 xenograft mice, and proteins were immunoblotted with the indicated antibodies. Relative intensity was calculated as described above (**A**).

The p53 tumor suppressor protein is a transcription factor that positively regulates the expression of genes involved in apoptosis and anti-proliferation of cancer cells [20,21]. We have previously reported that TNFα induces stabilization of p53 through the dissociation of the HDAC3-ERα complex, leading to apoptotic cell death exclusively in ERα-positive MCF7 cells [14]. Thus, we examined whether NCOR1 regulates p53 stabilization specifically in ERα-positive breast cancer cells. In MCF7 cells, p53 stabilization occurred to a similar extent upon *NCOR1* knockdown and TNFα treatment, whereas these alterations did not affect p53 protein levels in MDA-MB-231 cells. Moreover, an increased level or cleavage of the pro-apoptotic markers BAX and PARP-1, together with a decreased level of the anti-apoptotic marker Bcl-2, only appeared in MCF7 cells but not in MDA-MB-231 cells (Figure 3A). Consistently, in tumors isolated from MCF7 xenograft mice, TNFα induced p53 stabilization and NCOR1 destabilization, while *NCOR1* knockdown also led to the stabilization of p53 (Figure 3B). These results suggest that NCOR1 negatively regulates TNFα-induced p53 stabilization in ERα-positive breast cancer cells.

### 2.4. Knockdown of NCOR1 Activates the p21 Promoter via Recruitment of p53 Instead of ERα

**Figure 4:**
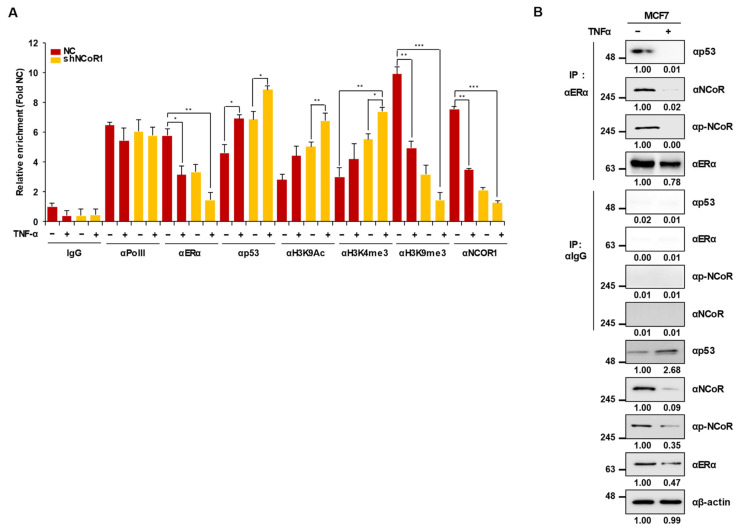
NCOR1 negatively regulates p53 recruitment to the *p21* promoter region and dissociates from the NCOR1-ERα-p53 complex upon TNFα treatment. (**A**) *NCOR1* knockdown or TNFα treatment resulted in the recruitment of p53 to the p53-binding element located between -360 and -260 bp of the *p21* promoter in MCF7 xenograft models. A chromatin immunoprecipitation (ChIP) assay was performed on the *p21* promoter region surrounding the p53-binding element (−360 to −260 bp) using tumors from MCF7 xenograft mice. Intensities of protein bands obtained from the immunoblotting assay were quantified with ImageJ and normalized with respect to that of β-actin. Relative intensity was calculated by the normalized control intensity of each protein. * *p* < 0.05, ** *p* < 0.01, and *** *p* < 0.001; Student’s *t*-test. The values presented are the means ± SD of three independent experiments. (**B**) The NCOR1-p53-ERα complex is dissociated upon TNFα treatment of MCF7 cells. A co-immunoprecipitation (Co-IP) assay was conducted using an anti-ERα antibody in MCF7 cell lysates with or without TNFα treatment. Proteins were immunoblotted with the indicated antibodies.

In our previous study, we showed that TNFα induces ERα degradation and subsequent p53 stabilization and recruitment to its target gene promoter [14]. However, interestingly, *NCOR1* knockdown did not reduce ERα protein levels, although TNFα treatment resulted in the marked degradation of ERα in both MCF7 cells (Figure 3A) and tumors from MCF7 xenograft mice (Figure 3B). Thus, we examined whether *NCOR1* knockdown reduced ERα occupancy of the *p21* promoter without affecting the ERα protein level. To this purpose, a chromatin immunoprecipitation (ChIP) assay was carried out with the *p21* promoter region including the p53-binding element (−360 to −260 bp) in tumors isolated from MCF7 xenograft mice. Interestingly, *NCOR1* knockdown impaired the binding of ERα to the promoter while enhancing that of p53. Notably, there were no significant differences between TNFα-treated and *NCOR1*-silenced groups. Further, both *NCOR1* knockdown and TNFα treatment resulted in enhanced H3K9ac-to-H3K9me3 transition and an increased level of H3K4me3 at the *p21* promoter region, denoting an active promoter state (Figure 4A). In addition, a co-immunoprecipitation (Co-IP) assay revealed that the NCOR1-ERα-p53 complex was dissociated upon TNFα treatment of MCF7 cells (Figure 4B). Collectively, these results demonstrate that NCOR1 is a negative regulator of TNFα-induced p53 stabilization and recruitment to the activated *p21* promoter region.

### 2.5. Tamoxifen Sensitivity Is Enhanced by TNFα or NCOR1 Knockdown in the MCF7 Xenograft Mouse Model

**Figure 5:**
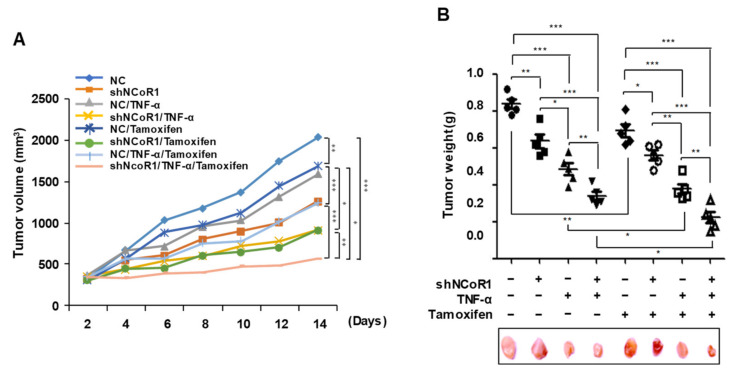
TNFα treatment or *NCOR1* knockdown enhances tamoxifen sensitivity in the MCF7 xenograft model. (**A**,**B**) TNFα treatment or *NCOR1* knockdown promotes tamoxifen sensitivity of tumor cells in the MCF7 xenograft mouse model. MCF7 cells infected with sh-Control or sh-NCOR1 were implanted into the subcutaneous space of mice, and Doxycycline (Dox) or/and TNFα were administered every other day until necropsy. Tumor volumes were measured every 2 days for 14 days, and representative images were captured. Data are expressed as means (*n* = 5 for each group) (**A**). All tumors were removed for necropsy, during which tumor weight was measured. All data are expressed as means ± SD (*n* = 5 for each group). * *p* < 0.05, ** *p* < 0.01, and *** *p* < 0.001; Student’s *t*-test (**B**). The values presented are the means ± SD of three independent experiments.

Tamoxifen is a drug that antagonizes ER and induces apoptosis in ER-positive breast cancer cells [22]. However, tamoxifen shows limitations as a single agent because its long-term use can result in the development of endocrine therapy resistance [23,24]. Therefore, to evaluate whether combining tamoxifen with TNFα treatment or suppression of *NCOR1* can potentially overcome tamoxifen resistance by enhancing the drug sensitivity of tumor cells, TNFα treatment or *NCOR1* knockdown were conducted in the presence or absence of tamoxifen in MCF7 xenograft mice for 14 days. Tumor volume was measured every 2 days for 14 days. Interestingly, the tumor volume was reduced by tamoxifen as well as TNFα treatment or *NCOR1* knockdown. Notably, combinatorial treatment with tamoxifen and TNFα or sh-NCOR1 decreased the tumor volumes more consistently than TNFα or sh-NCOR1 treatment alone (Figure 5A). Additionally, tumor weight was significantly decreased by combined treatment with tamoxifen and TNFα or sh-NCOR1 compared to single treatment with TNFα or sh-NCOR1 14 days after inoculation of tumor cells (*p* < 0.05) (Figure 5B). These results suggest that TNFα treatment or knockdown of *NCOR1*, its potent downstream regulator, enhances tamoxifen sensitivity of tumor cells in vivo.

## 3. Discussion

**Figure 6:**
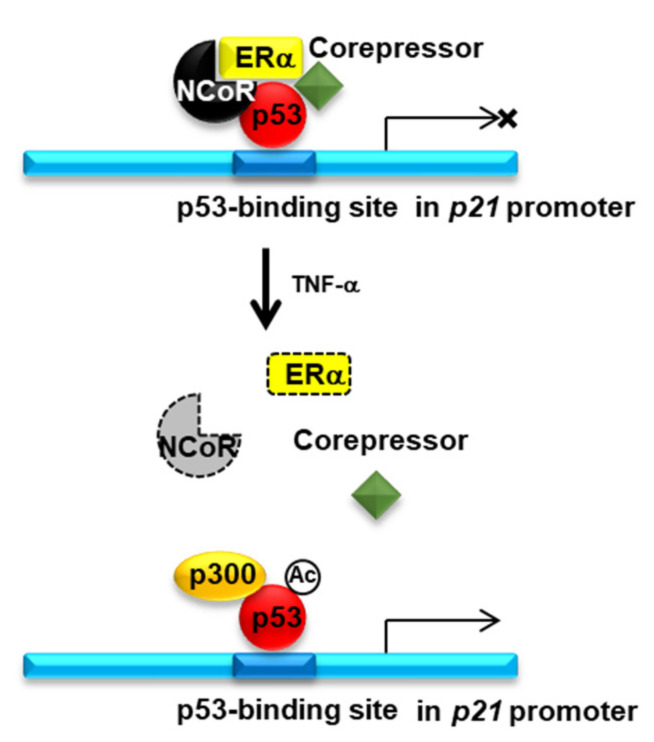
The model is suggested by the findings of the present study. The P53 forms a complex with NCoR1, ERα, and other corepressors on the p53-binding site in *p21* promoter region, and the transcriptional activity of p53 is repressed. Upon TNFα treatment, NCOR1 is destabilized and degraded, resulting in dissociation of the NCoR1-ERα-p53 complex. The p53 is further stabilized and transcriptionally activated.

Tamoxifen is a selective estrogen receptor modulator used to treat hormone receptor-positive breast cancer [25]. The efficacy and safety of tamoxifen monotherapy in breast cancer are comparable with those of many other endocrine treatments [26,27,28] and aromatase inhibitors [29,30]. However, previous studies have indicated the advantage of combinatorial treatment with tamoxifen and other drugs over tamoxifen treatment alone, since cancer patients showed a better response when tamoxifen was co-administered with drugs directed towards other molecular targets [31,32,33,34]. For instance, TNFα is a multifunctional cytokine involved in cellular signal transduction of various signaling pathways, such as those regulating apoptosis and cell survival as well as inflammatory responses and immunity [35]. Notably, many reports highlighted the antitumor effect of TNFα as well as its involvement in a wide spectrum of other diseases [36]. For example, our recent study showed that TNFα selectively induces apoptosis in ERα-positive MCF7 breast cancer cells [14], suggesting that this protein can be potentially combined with tamoxifen in cancer chemotherapy. In the present study, we demonstrated that TNFα specifically enhances tamoxifen efficacy via NCOR1 to suppress the growth of ERα-positive breast cancer (Figure 6).

NCOR1 is an important component of a transcriptional complex involved in the transcriptional repression of various genes [37] and is also known as a co-repressor of ERα [19,38]. Thus, we initially examined that whether NCOR1 expression is specifically regulated by TNFα in MCF7 ERα-positive breast cancer cells. Consistent with the specific modulation of ERα levels by TNFα in MCF7 cells [14], NCOR1 protein levels were decreased by TNFα treatment only in MCF7 cells but not in ERα-negative MDA-MB-231 cells. Thus, we hypothesized that NCOR1 is associated with the specific antitumor effect of TNFα. Indeed, the role of NCOR1 in cell proliferation and cancer development has been described in previous reports [39,40,41,42], and our data showed that *NCOR1* knockdown selectively suppressed the proliferation of ERα-positive cells in vitro and tumor growth in vivo. Consistently with the specific pro-proliferative function of NCOR1, *NCOR1* knockdown induced apoptosis selectively in ERα-positive but not in ERα-negative breast cancer cells. Moreover, we observed specific TNFα-induced apoptosis of ERα-positive cells, consistently with our previous report [14], and we further confirmed that knockdown of *NCOR1* and TNFα treatment exerted a similar targeted apoptosis-inducing effect in vitro and tumor growth inhibition in vivo. Therefore, these results support the hypothesis that NCOR1 is associated with the antitumor activity of TNFα in ERα-positive breast cancer.

Stabilization of the tumor suppressor protein p53 is an important mechanism promoting p53-mediated apoptosis of cancer cells and is regulated by various factors, such as the presence of E3 ligase, DNA damage, and cytokines [43,44,45]. In our previous study, we revealed that stabilization of p53 is a key mechanism for TNFα-induced specific cell death in ERα-positive breast cancer [14]. Thus, we assumed that NCOR1 negatively regulates p53 stabilization in ERα-positive cells. As expected, p53 stabilization occurred specifically in MCF7 cells upon both TNFα treatment and *NCOR1* knockdown. In addition, combined treatment with TNFα and sh-NCOR1 resulted in an additive effect on p53 stabilization. Various TNFα-dependent mechanisms, such as ERα degradation followed by HDAC inactivation [14] or NCOR1 degradation (present study), may participate in the regulation of p53 stabilization. Interestingly, *NCOR1* knockdown did not induce ERα degradation in either MCF7 cells or tumors isolated from MCF7 xenograft mice, indicating that other mechanisms may be associated with NCOR1-dependent negative regulation of p53 stabilization. Interestingly, our ChIP assay showed that *NCOR1* knockdown decreased ERα occupancy of the *p21* promoter region while increasing that of p53, thereby inducing an active promoter status; this effect corresponded to that of TNFα treatment but without alterations of ERα levels. Konduri et al. (2010) have reported that NCOR1, SMRT, p53, HDAC1, and ERα coexist in a complex on the p53-binding site of the *p21* promoter [46]. Consistent with this previous study, our Co-IP results showed that ERα forms a complex with NCOR1 and p53, and revealed that the complex was dissociated by TNFα treatment via NCOR1 degradation. Collectively, these results suggest that TNFα selectively induces dissociation of the NCOR1-p53-ERα complex and converts the *p21* promoter to its active state by recruiting p53 in ERα-positive breast cancer cells.

Tamoxifen resistance or tolerance can be acquired by ER-positive breast cancer patients through various mechanisms, such as modulation of ER signaling, upregulation of growth factor biosynthesis, and activation of the AKT/mTOR pathway [12,47]. Notably, binding of tamoxifen to ER recruits ER co-repressors such as NCOR1, which repress gene expression [48]. We found that NCOR1, complexed with ERα and p53, represses p53 transcriptional activity by interfering with its binding on the *p21* promoter, whereas the dissociation of this complex in presence of TNFα resulted in p53 recruitment and activation of the *p21* promoter. Thus, we expected that TNFα treatment or *NCOR1* knockdown contributed to overcoming tamoxifen tolerance by increasing the sensitivity of cancer cells to this drug. Indeed, in MCF7 xenograft mice, combined treatment with tamoxifen and TNFα or *NCOR1* knockdown reduced tumor volume and weight compared to TNFα treatment or *NCOR1* knockdown alone, suggesting that tamoxifen sensitivity is enhanced by TNFα and that NCOR1 is an important factor acting downstream of TNFα for overcoming tamoxifen resistance in vivo. However, it is huge limitation that only one cell line, MCF7, was used as a ERα-positive model. Thus, to firmly solidify our hypothesis, further verification using various ERα-positive models should be conducted.

## 4. Materials and Methods

### 4.1. Cell Culture Conditions, Reagents, and Antibodies

Human breast cancer MCF7 and MDA-MB-231 cells were obtained from the American Type Culture Collection (Manassas, VA, USA) and cultured in MEM supplemented with 10% fetal bovine serum (FBS; Gibco-BRL, MD, USA) and 1% antibiotic–antimycotic solution (Waltham, MA, USA) in a humidified 5% CO_2_ atmosphere at 37 °C. The antibodies used in this study is listed in Table S1 (Supplementary Information).

### 4.2. Western Blot

Protein extracts were prepared from TNFα-treated cells, and the expression of PARP-1, Bax, Bcl-2, and β-actin was determined by Western blot. MCF7 and MDA-MB-231 cells were treated with TNFα. Cells were collected 24 h after treatment, washed once with PBS, and extracts were prepared with lysis buffer (50 mM Tris-Cl (pH 7.5), 150 mM NaCl, 1% NP40, 10 mM NaF, 10 mM sodium pyrophosphate, and protease inhibitors). Protein extracts were separated using 10% SDS-polyacrylamide gels and transferred to nitrocellulose membranes. Blots were blocked by incubation with blocking solution for 1 h, incubated with the primary antibody for 2 h at room temperature or overnight at 4 °C, and finally processed with HRP-conjugated secondary antibody. Protein bands were visualized using the FUSION-SOLO imaging system (Vilber Lourmat, ZAC de Lamirault, France).

### 4.3. Quantification of Apoptotic Cells by Flow Cytometry

Apoptotic cells were quantified using the BD Pharmingen™ PE Annexin V Apoptosis Detection kit (BD Biosciences, San Jose, CA, USA). MCF7 and MDA-MB-231 cells were treated with TNFα. Cells were collected 24 h post-treatment and incubated for 15 min with the annexin V-PE antibody and propidium iodide in 1× binding buffer. Next, the apoptotic cell population (annexin-positive) was analyzed using a BD FACSCalibur™ flow cytometer (BD Biosciences), and results were analyzed using ModFit LT 2.0 (Verity Software House, Inc., St. Lexington, ME, USA).

### 4.4. Cell Proliferation Measurement by MTT Assay

The proliferation of MCF7 and MDA-MB-231 cells was determined using conventional MTT reduction assays. Briefly, cells (4000 cells/well) were plated in 96-well, white-walled, clear-bottom plates and incubated for 24 h at 37 °C. Cells were treated with vehicle (DMSO) and increasing concentrations of TNFα (0, 50, 100, and 200 μg/mL). After 24, 48, and 72 h, 100 μL of assay reagent was added to each well. The plate was incubated in the dark for 15 min, and luminescence was measured using a SpectraMax 250. All MTT assay data are presented as mean ± SD of three independent experiments.

### 4.5. Lentiviral Short Hairpin RNAs

We used shRNA-mediated silencing to establish stable MCF7 and MDA-MB-231 cell lines with reduced *NCOR1* expression. First, two pairs of commercially available oligonucleotides encoding each target-specific shRNA were purchased (MISSION^®^ shRNA, Sigma-Aldrich, St. Louis, MO, USA). Next, we prepared lentiviral particles producing pLKO.1-PURO plasmids containing shRNA, using co-transfection with three plasmids according to the manufacturer’s instructions (Invitrogen, Carlsbad, CA, USA). In brief, the plasmids were co-transfected into 293FT cells, which were then incubated for 3 days. Afterward, transfected cells were selected with puromycin, and lentivirus was isolated from the culture medium and concentrated with a Centricon^®^ Plus-20 Centrifugal Filter Unit (Millipore, Burlington, MA, USA). Further, either MCF7 or MDA-MB-231 cells were infected with lentiviruses expressing si-NCOR1 or PURO shRNA as a control.

### 4.6. Chromatin Immunoprecipitation Assays

Chromatin immunoprecipitation (ChIP) assays were performed with the indicated antibodies according to the manufacturer’s instruction (Promega, Madison, WI, USA). Eluted DNA was amplified with specific primers using SYBR™ green PCR master mix (Applied Biosystems, Foster City, CA, USA). The antibodies and primers used for ChIP assays are listed in Table S1 or Table S2, respectively (Supplementary Information).

### 4.7. Colony Formation Assay

Cells were suspended in MEM medium containing 10% FBS and 0.3% SeaKem^®^ LE agarose (Lonza, Basel, Swiss). A total 1 × 10^3^ cells in a volume of 15 mL were plated in six-well plates over a 1.5-mL layer of solidified MEM containing 10% FBS and 0.6% agarose. The plates were incubated at 37 °C for 3 weeks, and then the colonies formed on each plate were photographed from three different sides. The number and sizes of colonies on each plate were measured.

### 4.8. Generation of Dox-Inducible Stable Cell Lines

To manufacture NcoR1 knockdown stable cells, MCF7 and MDA-MB-231 cells were infected with Lentivirus carrying pLKO-TetON control shRNA and pLKO-TetON NcoR1 shRNA plasmids, and stable cells were selected by puromycin (5 μg/mL). NcoR1 knockdown induced in the presence of Doxycycline 100 ng/mL at 48 h.

### 4.9. Xenograft Mouse Model

The protocol for the care and used of animals were approved by the Asan Medical Center (AMC) SPF facility of the University of Ulsan College of Medicine (2020-02-222, 18 August 2020) in accordance with the International Animal Care and Use Committee (IACUC) guidelines. The tumor weight and survival were observed by using MCF7 cells, MDA-MB-231 cells, or pLKO-TetON NcoR1 shRNA-expressing MCF7 cells injected xenograft mouse model. MCF7 cells are estrogen-dependent in mice, Female, 6-week-old athymic, inbred BALB/c-nu mice. Mice were implanted with β-estradiol pellets (0.72 mg/pellet, 60-day release), purchased from Sigma-Aldrich (E8875, Saint Louis, MO, USA) into the dorsal shoulder blade region of mice. 10 mice in each experimental group were injected with 6 × 10^6^ cells resuspended in 100 μL PBS into the subcutaneous space of the left or right flank of mice, respectively. We injected tamoxifen pellets (5 mg/pellets, 60-day release) into Mice to deliver tamoxifen. All tumors were collected 2–3 weeks after inoculation for determination of tumor weight and volume by necropsy. Tumor diameters were measured 3 times a week, and tumor volumes were also calculated (volume = X × Y × Z × π/6).

### 4.10. Statistical Analyses

Statistical analyses were performed using Student’s *t*-test or two-way ANOVA and the SPSS software (Chicago, IL, USA). A *p* < 0.05 was considered as the threshold for statistical significance.

## 5. Conclusions

In conclusion, we have elucidated that TNFα increases tamoxifen sensitivity through dissociation of the ERα-p53-NCOR1 complex in ERα-positive breast cancer cells. Furthermore, we presented NCOR1 as a key regulator associated with TNFα-dependent modulation of tamoxifen sensitivity. Altogether, our results indicated that pharmacological or genetic disruption of NCOR1 could be an efficient targeted therapy for overcoming tamoxifen resistance in ERα-positive breast cancer.

## Data Availability

Not applicable.

## Supplementary Materials

The following are available online at https://www.mdpi.com/article/10.3390/cancers13112601/s1, Figure S1. Analysis of the association between NCOR1 and P53 in ER+ breast cancer; Table S1. Antibodies used for WB and ChIP assays; Table S2. Primers used for qRT-PCR and ChIP assays; Table S3. *NCOR1* shRNA sequences for knockdown system.
