# Supplementary material for: TNFα Enhances Tamoxifen Sensitivity through Dissociation of ERα-p53-NCOR1 Complexes in ERα-Positive Breast Cancer
Source: Cancers (Basel). 2021 May 26;13(11):2601. doi: 10.3390/cancers13112601 (PMC8199199; doi:10.3390/cancers13112601)
Supplement: Supplementary file 1 [file cancers-13-02601-s001.zip › cancers-1211431-supplementary conversion.pdf]

cancers MDPI

Supplementary Materials: TNFα Enhances Tamoxifen Sensitivity through Dissociation of ERα-p53-NCOR1 Complexes in ERα-Positive Breast Cancer

Hyunhee Kim, Seung-Ho Park, Jangho Lee, Gi-Jun Sung, Ji-Hye Song, Sungmin Kwak, Ji-Hoon Jeong, Min-Jeong Kong, Jin-Taek Hwang, Hyo-Kyoung Choi and Kyung-Chul Choi

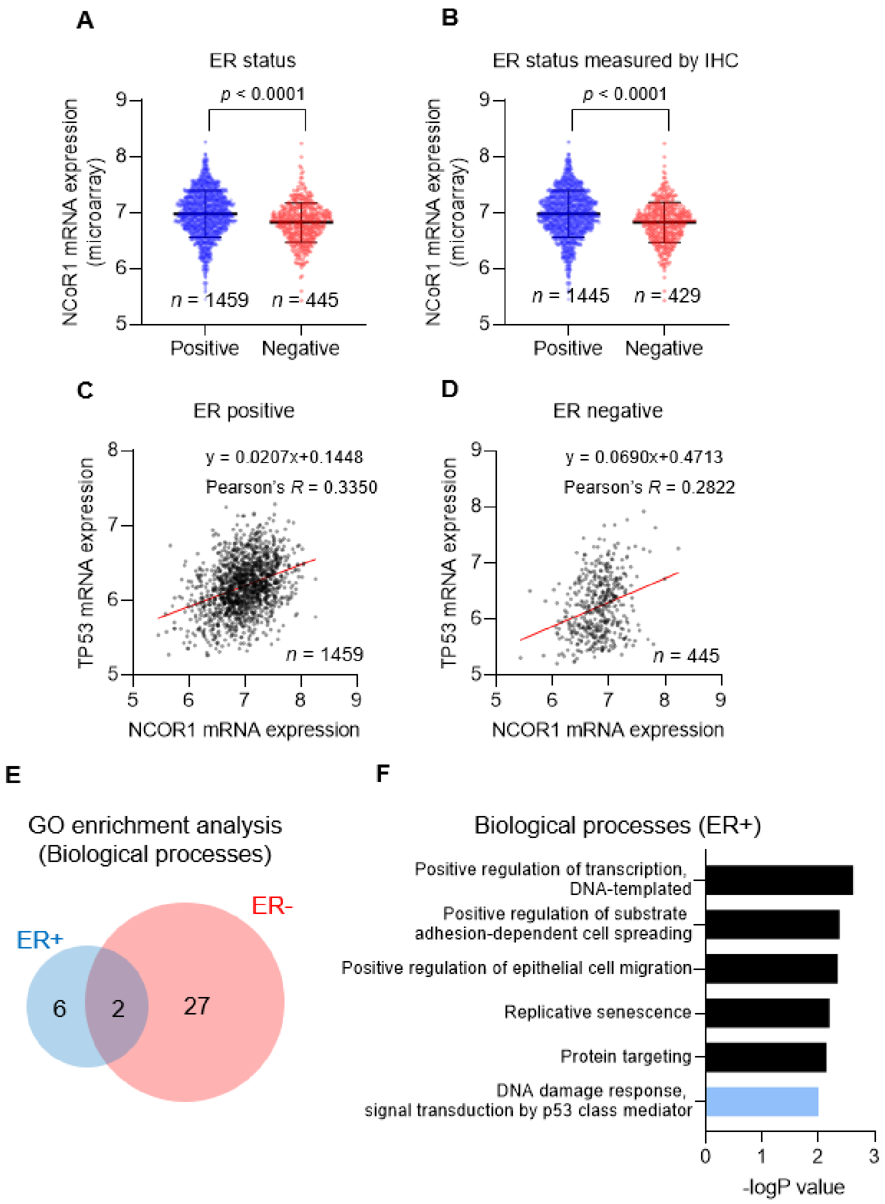

Figure S1. Analysis of the association between NCOR1 and P53 in ER+ breast cancer. The association between NCOR1 and P53 signaling in ER+ breast cancer. The transcriptome data of breast cancer cohort from The Cancer Genome Atlas (TCGA) (n = 1,904) was analyzed. (A, B) *Ncor1* mRNA levels in ER positive or ER negative breast cancer. ER status was measured by RNA-Seq (A) or IHC (B). (C, D) The correlation between *Ncor1* and *Tp53* mRNA expressions in ER positive (E) or ER negative breast cancer (E). Venn diagram for the biological processes enriched in ER+ and/or ER− breast tumors. (F) The biological processes enriched only in ER+ breast tumor.

Table S1. Antibodies used for WB and ChIP assays.

Antibodies	Vendor<br>Cat no.	Local	Country	Titer	Usage
αH3K9ac	Merck<br>06-942	Darmstadt	Germany	(1:100)	W. B<br>(ChIP)
αH3K4me3	Merck<br>05-1339	Darmstadt	Germany	(1:100)	
αH3K9me3	Merck<br>05-1250	Darmstadt	Germany	(1:100)	
αERa	Santa Cruz Biotechnol-ogy<br>SC-543	Dallas, Texas	USA	1:1000<br>(1:100)	
αp53	Santa Cruz Biotechnol-ogy<br>SC-126	Dallas, Texas	USA	1:1000<br>(1:100)	
αp-p53	Abcam<br>ab134190	Cambridge	UK	1:1000	W.B
αNCoR	ATGEN	Seongnam, Gyeonggido	Korea	1:1000	
αp-NCoR	ATGEN	Seongnam, Gyeonggido	Korea	1:1000	
PARP-1	Cell signaling<br>9542S	Danvers, MA	USA	1:1000	
Bax	Cell signaling<br>5023S	Danvers, MA	USA	1:1000	
Bcl-2	Santa Cruz Biotechnol-ogy<br>SC-492	Dallas, Texas	USA	1:1000	
αβ-Actin	Sigma-Aldrich<br>A5441	Saint Louis, MO	USA	1:10000	

Abbreviation: αH3K9ac, anti-H3K9 acetylation; αH3K4me3, anti-H3K4 trimethylation; αH3K9me3, anti-H3K9 trimethylation; αERα, anti-Estrogen Receptor α; αp53, anti-p53; αp-p53, anti-phospho-p53; αNCoR, anti-Nuclear receptor co-repressor; α-PARP-1, anti-PARP-1; αBax, anti-Bax αBcl-2, anti-Bcl-2; αβ-Actin, anti-beta Actin; W.B, Western blotting; ChIP, Chromatin immunoprecipitation.

Table S2. Primers used for qRT-PCR and ChIP assays.

Primer List			
NCoR1	Forward	5′ ACACCGCAGTATTGTCCAAAT 3′	qRT-PCR for human
	Reverse	5′ CACCTGGTTTGTCTTGATGTTCT 3′	
GAPDH	Forward	5′ TGATGACATCAAGAAGGTGGTGAAG 3′	
	Reverse	5′ TCCTTGGAGGCCATGTAGGCCAT 3′	
p21 promoter	Forward	5′ GTAAATCCTTGCCTGCCAGAG 3′	qPCR for ChIP assay
	Reverse	5′ GCGCTGGACACATTTCCCCA 3′	

Abbreviation: NCoR1, Nuclear receptor co-repressor1; GAPDH, glyceraldehyde-3-phosphate dehydrogenase; p21, cyclin-dependent kinase inhibitor 1; ChIP assay, chromatin immunoprecipitation assay.

Table S3. *NCOR1* shRNA sequences for knockdown system.

	**shRNA sequences list**
shNCoR1 #1	5′ CCGGGCCCACAGATGATGAAGAAATCTCGAGATTTCTT CATCATCTGTGGGCTTTTTG 3′
shNCoR1 #2	5′ CCGGGCCATCAAACACAATGTCAAACTCGAGTTTGACA TTGTGTTTGATGGCTTTTTG 3′

Abbreviation: NCoR1, Nuclear receptor co-repressor1.
