# Supplementary figures and images for: TNFα Enhances Tamoxifen Sensitivity through Dissociation of ERα-p53-NCOR1 Complexes in ERα-Positive Breast Cancer
Source: Cancers (Basel). 2021 May 26;13(11):2601. doi: 10.3390/cancers13112601 (PMC8199199; doi:10.3390/cancers13112601)

Figure 1.

A

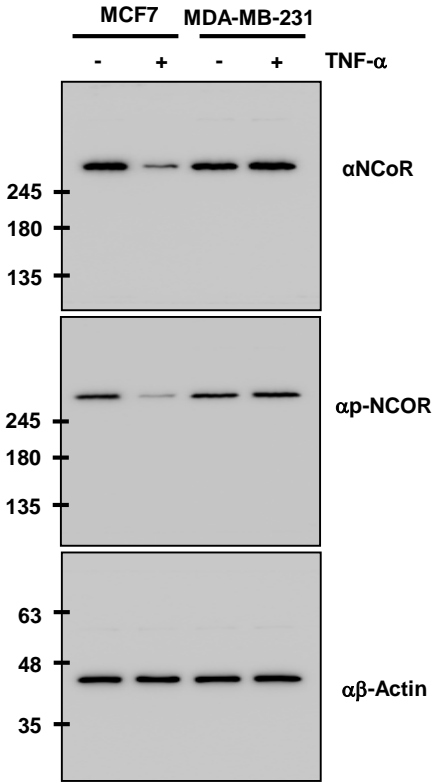

B

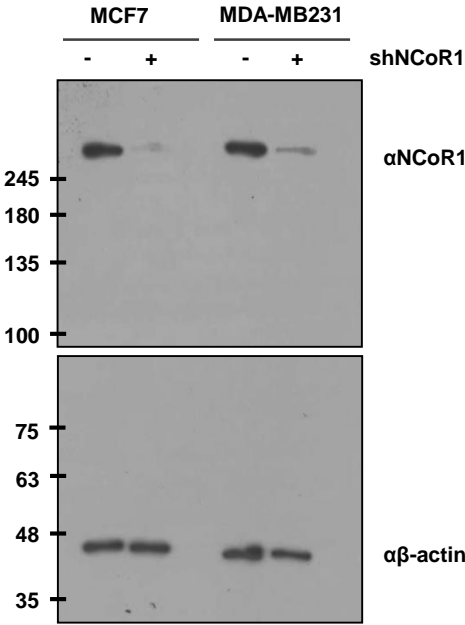

Figure 3.

A

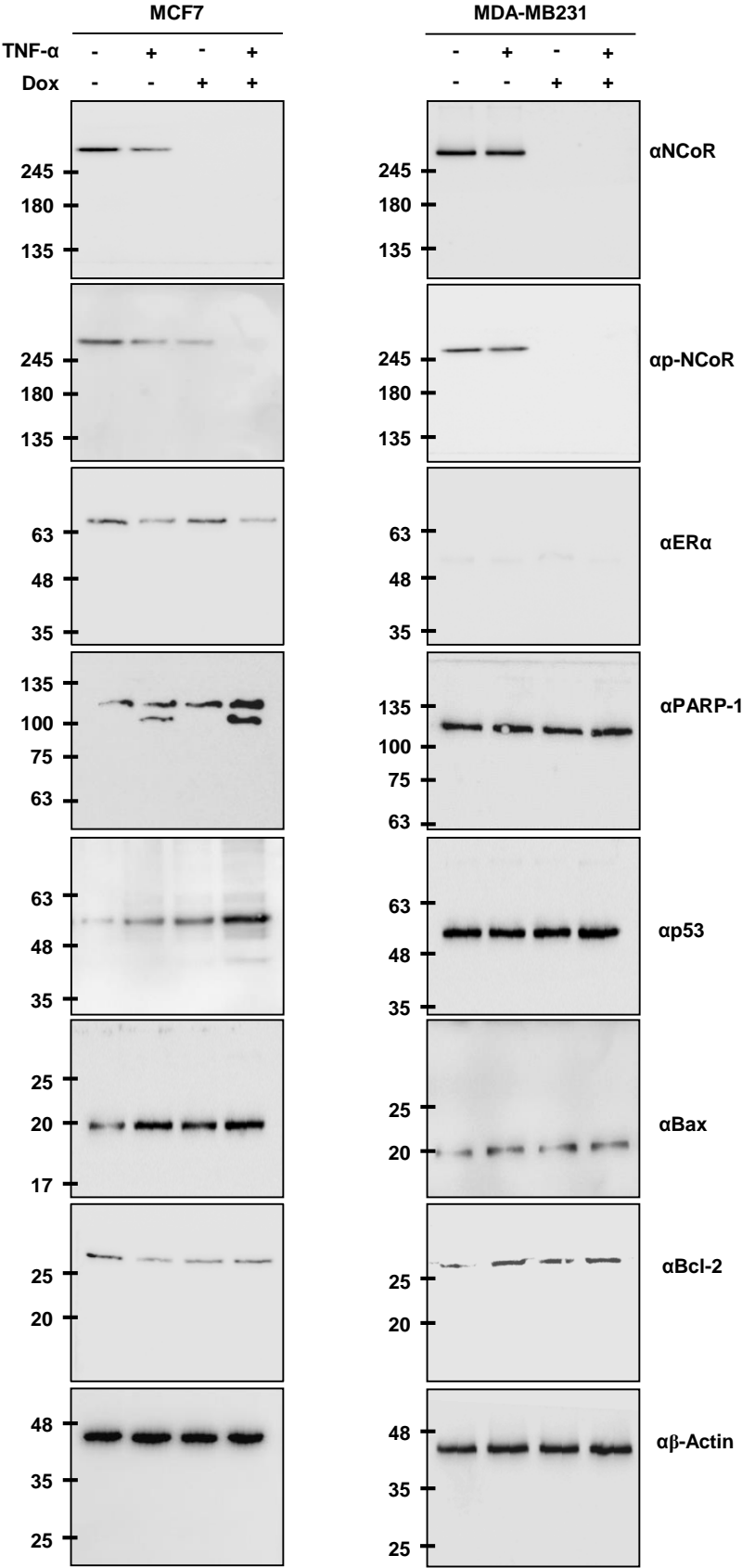

Figure 3.

B

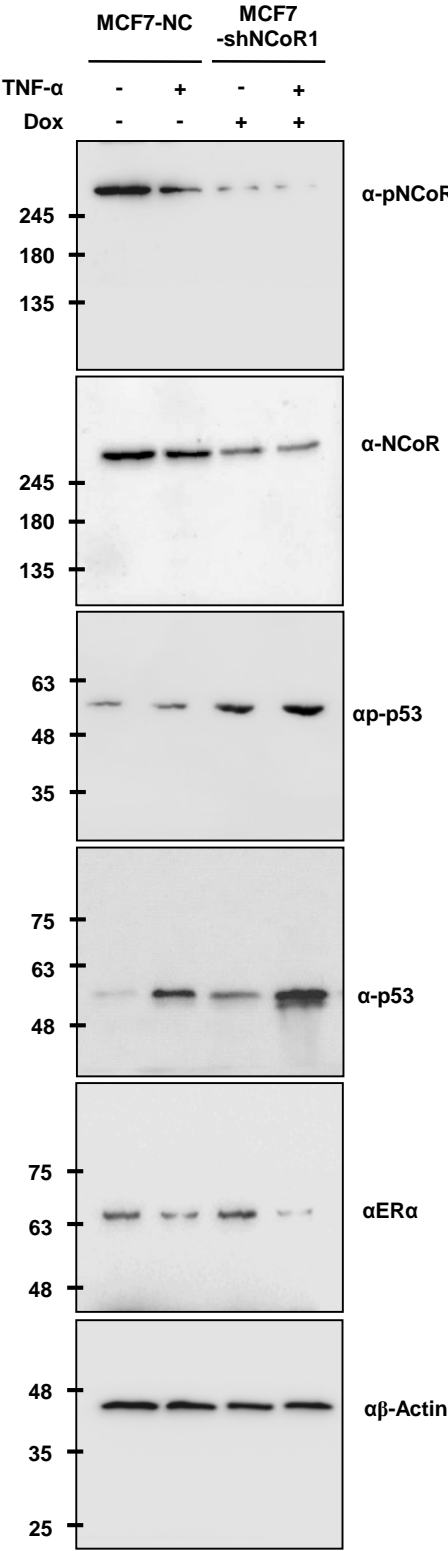

Figure 4.

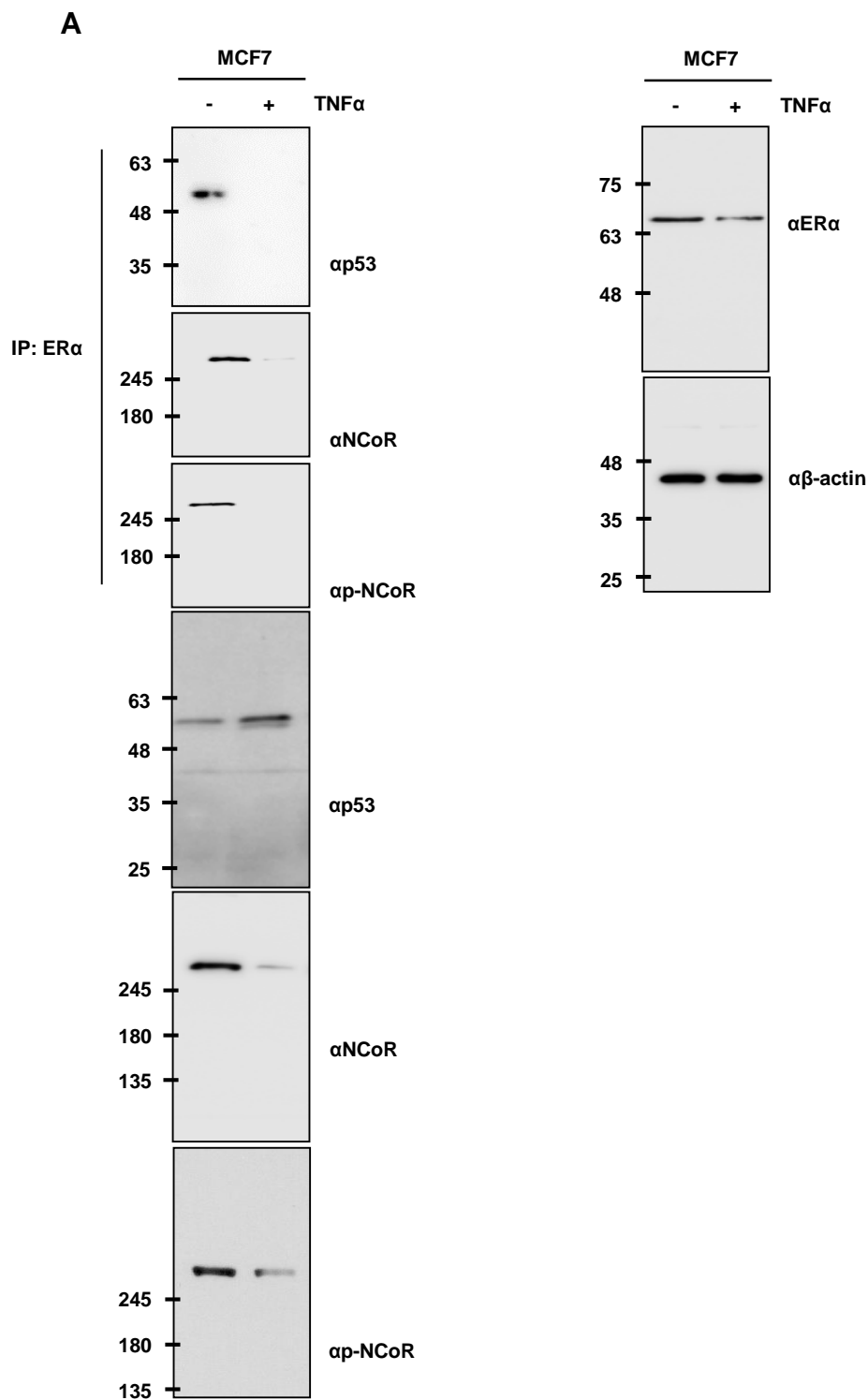

Supplement: Supplementary file 1 [file cancers-13-02601-s001.zip › cancers-1211431-WB.pdf]
